# Decoding Adaptive Visuomotor Behavior Mediated by Non-linear Phase Coupling in Macaque Area MT

**DOI:** 10.3389/fnins.2020.00230

**Published:** 2020-04-03

**Authors:** Mohammad Bagher Khamechian, Mohammad Reza Daliri

**Affiliations:** ^1^Neuroscience and Neuroengineering Research Lab, Biomedical Engineering Department, School of Electrical Engineering, Iran University of Science & Technology, Tehran, Iran; ^2^Cognitive Neurobiology Laboratory, School of Cognitive Sciences (SCS), Institute for Research in Fundamental Sciences (IPM), Tehran, Iran

**Keywords:** bicoherence, quadratic phase coupling, non-linear phase synchronization, visual area MT, spatial attention

## Abstract

The idea that a flexible behavior relies on synchronous neural activity within intra- and inter-associated cortical areas has been a matter of intense research in human and animal neuroscience. The neurophysiological mechanisms underlying this behavioral correlate of the synchronous activity are still unknown. It has been suggested that the strength of neural synchrony at the level of population is an important neural code to guide an efficient transformation of the sensory input into the behavioral action. In this study, we have examined the non-linear synchronization between neural ensembles in area MT of the macaque visual cortex by employing a non-linear cross-frequency coupling technique, namely bicoherence. We trained a macaque monkey to detect a brief change in the cued stimulus during a visuomotor detection task. The results show that the non-linear phase synchronization in the high-gamma frequency band (100–250 Hz) predicts the animal’s reaction time. The strength of non-linear phase synchronization is selective to the target stimulus location. In addition, the non-linearity characteristics of neural synchronization are selectively modulated when the monkey covertly attends to the stimulus inside the neuron’s receptive field. This additional evidence indicates that non-linear neuronal synchronization may be affected by a cognitive function like spatial attention. Our neural and behavioral observations reflect that two crucial processes may be involved in processing of visuomotor information in area MT: (I) a non-linear cortical process (measured by the bicoherence) and (II) a linear process (measured by the spectral power).

## Introduction

Neural oscillations are frequently observed in cortical activities. Notably, it has been widely asserted that neural oscillations are involved in many cortical computations, including sensory coding (Siegel et al., 2007; Belitski et al., 2008; Schroeder and Lakatos, 2009) and information transmission (Hipp et al., 2011; van Kerkoerle et al., 2014; Rohenkohl et al., 2018). Brain networks can communicate through frequency-specific oscillations. These oscillatory activities can play a functional role in brain networks to flexibly integrate, process, and transmit neural information among cortical circuitries (Moore and Armstrong, 2003; Buschman and Miller, 2007; Saalmann et al., 2007; Siegel et al., 2008; Hipp et al., 2011). However, recent studies have suggested that brain oscillations could interdependently interact, forming so-called cross-frequency coupling (CFC) (Buzsáki, 2006; Jensen and Colgin, 2007). This form of interactive computation has been observed in several brain areas of different species (Canolty et al., 2010; Igarashi et al., 2014; Esghaei et al., 2015). The CFC has an important role in many cortical functions, including sensory processing (Saleh et al., 2010), learning (Tort et al., 2009; Igarashi et al., 2014), memory (Axmacher et al., 2010), and attention (Esghaei et al., 2015; Spyropoulos et al., 2018). It is believed that CFC can functionally facilitate information coordination between neurons, simultaneously in time and space (Aru et al., 2015). Furthermore, recent studies in human and non-human primates have shown that CFC may serve as a potential physiological mechanism underlying intra-areal communication in the brain (Darvas et al., 2009; Canolty and Knight, 2010; Holz et al., 2010; Fiebelkorn et al., 2018). For example, a study on the visuospatial working memory in human indicated that CFC between oscillatory phases of theta (4–8 Hz) and gamma (50–70Hz) activities can regulate an effective communication between occipital and parietal brain regions (Holz et al., 2010). Another investigation in macaque monkey suggested that coupling between the phase of theta oscillations (3–8 Hz) and the power of high frequencies (9–45 Hz) during spatial attention potentially facilitates an interregional communication between the frontal eye field (FEF) area, lateral intraparietal area (LIP), and visual cortex (Fiebelkorn et al., 2018).

Contemporary investigations into visual areas have shown that oscillatory components of local field potential (LFP) (Liu and Newsome, 2006; Womelsdorf et al., 2006; Smith et al., 2015; Khamechian et al., 2019) and neural spiking activity (Liu and Newsome, 2005; Smith et al., 2015; Parto Dezfouli et al., 2018) could provide useful information about how neural activities are linked to visuomotor behavior. These studies have reported a trial-by-trial correlation between the power of beta (10–30 Hz) (Smith et al., 2015), gamma, and high-gamma (50–200 Hz) (Liu and Newsome, 2006) LFPs and behavioral output. Moreover, they have shown that the strength of gamma (Womelsdorf et al., 2006) and high-gamma synchronization (Khamechian et al., 2019) between sensory neurons in the dorsal and ventral visual pathway, respectively, predict the speed of behavioral responses. Despite these promising observations on neural-behavior correlation in the sensory visual areas, the contributive role of non-linear neuronal synchronization in guiding visuomotor behavior has not been studied in the visual cortex.

Bicoherence is an advanced signal processing technique capable of tracking the neuronal non-linearity and non-Gaussian signals underlying brain functions (Bullock et al., 1997; Darvas et al., 2009; Li et al., 2009, 2013). Many studies has shown that this technique can quantify the strength of non-linear phase-phase CFC [i.e., quadratic phase coupling (QPC)] between frequency components of the LFP signal (von Stein et al., 2000; Wang et al., 2007; Darvas et al., 2009; Sheremet et al., 2019). The neural generators of QPC have been reported for object coding in single neuron, in which different features of an object (e.g., size and angular speed) are encoded by a multiplicative process (Gabbiani et al., 2002). QPC has also been found in neuronal control circuits underlying sensorimotor control (Ahissar and Kleinfeld, 2003). Furthermore, QPC can effectively facilitate transmission of selective information between cortical networks (Darvas et al., 2009; Akam and Kullmann, 2014). On the other hand, it has been shown that the QPC plays a key role in multiplexing neural signals, which improves neural transmission (Akam and Kullmann, 2014).

Here, we have studied LFP signals by employing the bicoherence method to examine how non-linear neuronal synchronization in the MT area is involved in the processing of visuomotor information. For this purpose, we trained a macaque monkey to perform a visuomotor detection task. The animal had to detect a brief change in the target stimulus. Results have indicated that the strength of non-linear phase synchronization among MT neurons predicts the animal’s reaction time on a trial-by-trial basis. Importantly, we observed that the non-linear phase synchronization mostly occurs in the high-gamma frequency band (100–250 Hz) of LFPs, in line with a recent study (Khamechian et al., 2019). Moreover, the result demonstrated that non-linear characteristics of neuronal synchronization are modulated when the monkey covertly attends to the stimulus inside the neuron’s receptive field. Furthermore, we observed that the non-linear and the linear neuronal synchronizations potentially play a functional role in processing visuomotor information in the MT area of the visual cortex.

## Materials and Methods

### Animal Welfare

All animal procedures in this study were performed at the German Primate Center in Göttingen, Germany, and were approved by the responsible regional government office [Niedersaechsisches Landesamt fuer Verbraucherschutz und Lebensmittelsicherheit (LAVES)], under the permit numbers 33.42502/08-07.02 and 33.14.42502-04-064/07. For more details on the non-human primate facilities, training facilities, and surgical techniques in this laboratory, please see Roelfsema and Treue (2014), Calapai et al. (2017), Berger et al. (2018), Pfefferle et al. (2018).

### Experimental Task and Recording

**FIGURE 1:**
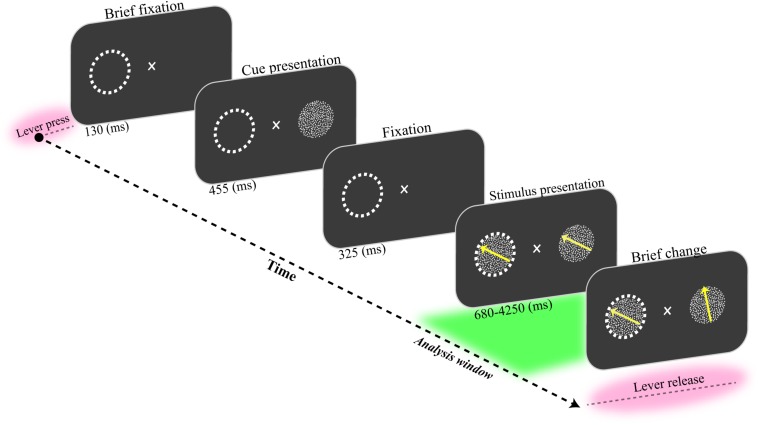
Behavioral paradigm. The monkey had to press a lever while maintaining his gaze on a fixation spot for 130 ms. Then, a static random dot pattern (RDP) appeared for 455 ms to indicate the upcoming target location on the screen. The screen was blanked for the next 325 ms. Next, two RDPs were presented inside and outside the receptive field (marked by a dashed circle here) for a random period of 680 to 4250 ms. The monkey received a drop of juice if it correctly detected a short change in the direction of the target RDP and released the lever within a short time window (150–650 ms). The analysis window was 1500 ms preceding the direction change in the target stimulus (delineated as a green area in the figure).

A male macaque monkey was trained to fixate on a central fixation point and covertly attend to one of two coherently moving random dot patterns (RDP). Each trial was initiated by pressing a lever while maintaining the gaze on a central fixation point for 130 ms (Figure 1). Next, a static RDP appeared for 455 ms to cue the upcoming target’s location. Following a short blank period (325 ms), two moving RDPs were shown inside and outside the receptive field (RF) of the recorded neurons for a random period of 680–4250 ms. The monkey had to release the lever immediately after the target underwent a brief change in direction of motion. The RDP’s direction for target and non-target (distractor) stimuli were the same, chosen randomly from eight possible directions (0–360° with steps of 45°). The monkey was rewarded if he correctly released the lever within 150–650 ms after the target change occurred. Trials were terminated without a reward when the monkey (i) broke the maintenance of his gaze on the fixation spot, (ii) released the lever in response to a distractor change, or (iii) responded too late after the target change. The monkey correctly detected the target changes in 86% of the trials without fixation breaks. He incorrectly terminated 3 and 11% of trials by responding to a non-target change (false alarm) and ended the trial without performing any response (miss trial), respectively.

Single-unit neural activities (SUAs) and local field potentials (LFPs) were recorded extracellularly from MT neurons using a multi-channel recording system (Mini-Matrix, Thomas Recording, and Plexon data acquisition system, Plexon Inc.). The signals were split into SUA and LFP by hardware filters. Moreover, the LFPs and the SUAs were amplified and digitized at 1 and 40 kHz, respectively. The 50 Hz noise of the power line was eliminated from the LFPs using a non-causal 4th-order Butterworth notch filter. Action potentials of recorded units were sorted online using a Plexon MAP data acquisition system (Plexon, Dallas, TX, United States). Single units were isolated online using a window discrimination procedure. The data were collected from 111 sites with five parallel electrodes, advanced separately into brain tissue to isolate direction-tuned MT neurons with overlapping RF. These electrodes were not implanted chronically but were inserted simultaneously in each experimental session. MT sites were identified by their anatomical location in cortex (using structural MRI imaging) and by the physiological properties of recorded neurons: neurons were direction-selective and the average diameter of the neuron’s RF was almost equal to the RF eccentricity. The RF centers of MT neurons at different locations were predictable along the superior temporal sulcus in cortex. For more details on the experimental procedure, behavioral task, and recording details see Esghaei and Daliri (2014).

### Data Analysis Procedure

In the following sections, the analyses and quantitative procedure are discussed. All analyses were implemented using MATLAB software (R2017b; MathWorks, Natick, MA, United States).

### Trial Selection Procedure

We only analyzed the hit trials in which the monkey correctly detected the target change. The hit trials were sorted based on reaction times (RTs) and sub-divided into four quartiles. An equal number of these trials were selected from the first and the last quartiles and labeled as the fast and the slow trials, respectively. Through this process, there were 725 trials at each fast and slow group. We used single-unit spiking activity and the LFP of chosen trials to predict the animal’s reaction time (RT). All analyses were carried out for stimulus presentation period, for a time window of 1500 ms before the target change occurred (see “Analysis window” in Figure 1). We chose the trials in which the target stimulus was changed 3000 ms after the trial onset. The rationale for this selection was to be ensured that the analysis window was far enough from the stimulus-evoked activities induced by the stimulus onset. We employed a built-in MATLAB function to perform digital filtering with zero-phase distortion (the *filtfilt* function).

### Analysis of Bicoherence

General harmonic wavelet transform (GHWT)-based wavelet bicoherence (WBIC) (Li et al., 2009, 2011) was used to measure the quadratic phase coupling (QPC) in LFP signals. A segment-averaging approach (Hagihira et al., 2001; Li et al., 2009) was employed for calculating WBIC in order to obtain a reliable estimate of bicoherence. We used a time window of 500 ms with a 375 ms overlap to divide the LFP signal into eight time epochs. For each epoch, the GHWT-based WBIC algorithm was run to calculate bicoherence in all frequency pairs from 1 to 250 Hz, with a step of 1 Hz and bandwidth of 2 Hz. The implementation of this algorithm is briefly explained in the following (for more details on the GHWT-based WBIC algorithm, see Li et al. (2009, 2011). First, we conducted the GWHT for each epoch of a trial’s LFP (*X*_*k*_(*t*), where *k* denotes the *k*th epoch of a given LFP signal) to calculate the wavelet coefficient *a*_*k*_(*f*,*t*) in a frequency component *f*. This frequency component varied from 1 to 250 Hz (as mentioned previously). Next, the normalized squared WBIC was calculated for each possible pair of frequency component as given in eq. 1:

$$b_{k}\,\left(f_{1},f_{2}\right)=\frac{{\left|B_{k}\,\left(f_{1},f_{2}\right)\right|}^{2}}{\sum_{t=1}^{N}{\left|a_{k}\,\left(f_{1},t\right)\,a_{k}\,\left(f_{2},t\right)\right|}^{2}\,\sum_{t=1}^{N}{\left|a_{k}\,\left(f_{1}+f_{2},t\right)\right|}^{2}},$$

(1) $$1\leq f_{1},f_{2}\leq 250\,\mathrm{Hz}$$

where *N* represents the time length of the epoch, (*f*_1_,*f*_2_) indicates a frequency pair (bifrequency), and *B*_*k*_ denotes the phase-randomized wavelet bicoherence, which is calculated as indicated in eq. 2:

(2) $$B_{k}\,\left(f_{1},f_{2}\right)=\sum_{t=1}^{N}a_{k}\,\left(f_{1},t\right)\,a_{k}\,\left(f_{2},t\right)\,a_{k}^{*}\,\left(f_{1}+f_{2},t\right)\,\,e^{i\,R\,\varphi_{k}\,\left(f_{1},f_{2},t\right)}$$

where *R* ∈ [−π,π] is a random variable and φ_*k*_(*f*_1_,*f*_2_,*t*) denotes instantaneous biphase, which is calculated using the function provided in eq. 3:

(3) $$\varphi_{k}\,\left(f_{1},f_{2},t\right)=\varphi_{k}\,\left(f_{1},t\right)+\varphi_{k}\,\left(f_{2},t\right)-\varphi_{k}\,\left(f_{1}+f_{2},t\right)$$

Next, we made use of a surrogate method to eliminate all spurious QPCs and obtained a reliable estimate for the wavelet bicoherence (Li et al., 2009, 2011). To this end, the biphase function φ_*k*_(*f*_1_,*f*_2_,*t*) was replaced with a new biphase $\varphi_{k}^{\prime }\,\left(f_{1},f_{2},t\right)=\varphi_{k}\,\left(f_{1},f_{2},t\right)+\theta$in Eq. 2 in order to calculate a surrogated bicoherence for a given bifrequency(*f*_1_,*f*_2_). *θ* is a random variable chosen from (−π,π]. We generated a hundred samples of surrogated bicoherence for the bifrequency(*f*_1_,*f*_2_) and computed their mean (μ) and standard deviation (σ). The original bicoherence was preserved if it exceeded μ + 1.6σof the surrogate bicoherence (as a 95% statistical threshold value); otherwise, it was set to zero. The GHWT-based WBIC method provided a two-dimensional bicoherence matrix with 250 × 250 bifrequency components for each trial’s LFP signal.

### Quantitative Analysis of the Bicoherence Matrix Using Bicoherence Indices

We calculated four indices using the bicoherence matrix obtained for each trial. These indices were computed in WBIC studies to quantify the bicoherence matrix (Li et al., 2009, 2011, 2013; Wang et al., 2017). They were computed for each trial’s bicoherence matrix as follows:

**(i)** Total amount of the wavelet bicoherence across all bifrequency pairs of (*f*_1_,*f*_2_);

(4) $$T\,o\,t\,a\,l\,B\,i\,c\,=\,\sum \sum b\,\,\left(f_{1},f_{2}\right)$$

where 1≤*f*_1_,*f*_2_≤250Hz and *b* is the bicoherence matrix.

**(ii - iii)** Eigen-decomposition for *b*; since bicoherence matrix is a symmetric matrix with respect to the main diagonal (*f*_1_ = *f*_2_), Eigen-decomposition can be conducted as follows:

(5) $$b\nu_{i}=\lambda_{i}\nu_{i},\lambda_{\text{i}}\in \left\{\lambda_{1}\leq \lambda_{2}\leq \ldots \leq \lambda_{M},M=250\right\}$$

where λ_*i*_,υ_*i*_are the eigenvalue and eigenvector, respectively. *M* denotes the number of frequency components (*f*). The maximum eigenvalue (Li et al., 2009) and Shannon entropy of the eigenvalue distribution (Cui et al., 2010; Dauwels et al., 2010; Li et al., 2011) were considered as the next bicoherence indices. The Shannon entropy of the eigenvalue distribution is computed with the following function:

(6) $$E\,n\,t\,r\,o\,p\,y\,\,o\,f\,\,e\,i\,g\,e\,n\,v\,a\,l\,u\,e\,s\,=-\frac{\sum_{i=1}^{M}\lambda_{i}^{\prime }\,\log\left(\lambda_{i}^{\prime }\right)}{\log\,\left(M\right)}$$

where $\lambda_{i}^{\prime }=\left|\lambda_{i}\right|/\sum_{i=1}^{M}\left|\lambda_{i}\right|$ is the normalized absolute eigenvalue.

**(iv)** Average diagonal elements of the bicoherence matrix (*f*_1_ = *f*_2_):

(7) $$D\,i\,a\,g\,o\,n\,a\,l\,B\,i\,c\,=\frac{\sum b\left(f_{1}=f_{2},f_{2}=f_{1}\right)}{M}$$

**FIGURE 2:**
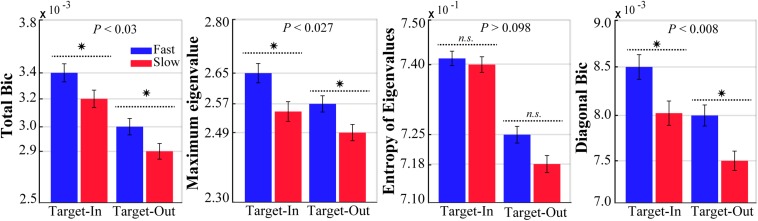
The bicoherence indices for the fast and the slow trials at each target location condition (Target-In\Target-Out). Stars show a significant difference between the bicoherence indexes calculated for the fast (blue) and the slow (red) trials (two-sided Wilcoxon rank-sum test, *P*-values have been reported on top of the bars). Error bars indicate SEM.

We computed the bicoherence indices for the fast and the slow trials in both target position conditions (target-in and target-out, see Figure 2). To ensure that the bicoherence indices were independent of the spectral power, a subset of the fast and the slow trials with no significant differences in their spectral power in a wide frequency band (0–500 Hz) were selected. We calculated the LFP band-power for each fast and slow trial in a wide frequency band (0–500 Hz). Consequently, the same number of trials were sub-selected from individual histogram bins of the fast and the slow band-power. This procedure provided two subsets of the fast and the slow trials that had no significant differences in power spectrums (*p* > 0.98, for both target stimulus conditions, using a two-sided Wilcoxon rank-sum test).

### Selection of the Bifrequency Components by Using a Feature-Ranking Method

**FIGURE 3:**
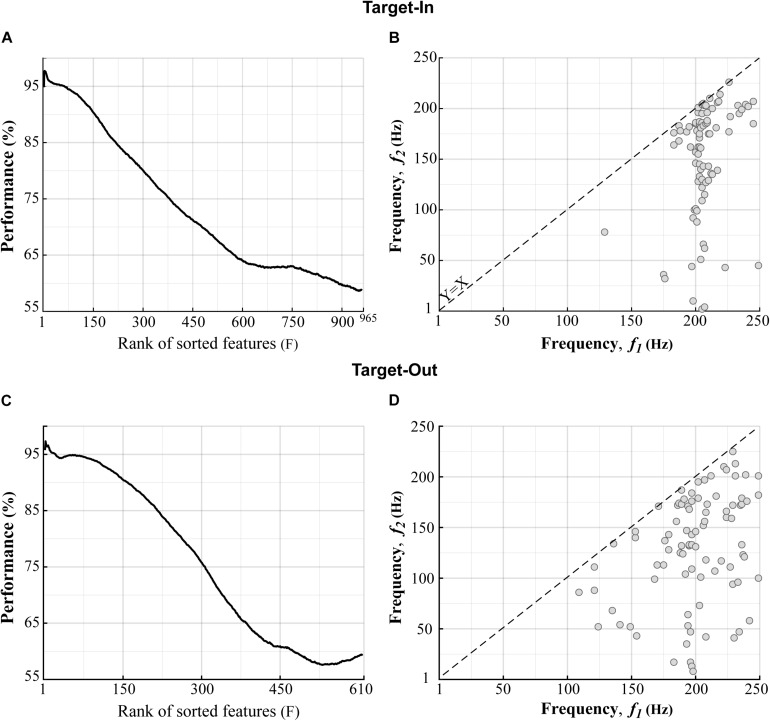
Distributions of the bifrequency components provided high classification performance in decoding the fast and the slow trials. **(A,C)** The bifrequency components (features) in the Target-In and the Target-Out conditions, respectively, which were sorted based on the decoding performance in descending order. **(B,D)** Distributions of the bifrequency components in panels **(A,C)**, respectively, which provided performance of over 90% in decoding the fast and the slow trials. **(B,D)** show 85 and 89 bifrequency components for the Target-In and the Target-Out conditions, respectively. We did not analyze the upper bound of the *X* = *Y*-axis in panels **(B,D)** because of the symmetric property of the bicoherence. *X*-*Y* axes indicate the center bound of frequency bands in panels **(B,D)**.

We compared the bifrequency components of the bicoherence matrix between the fast and the slow trials using a two-sided Wilcoxon rank-sum test. We then chose the bifrequency components with significance levels of *p* < 0.01. With this, 965 and 610 bifrequency components were sub-selected from 250 × 250 components in the bicoherence matrix for the target-in and the target-out conditions, respectively. The bicoherence at each bifrequency component was z-score-normalized across trials. We further employed a feature-ranking method to exclude bifrequency components yielding low performance in decoding the fast and slow trials. Firstly, a repeated holdout method was conducted for 100 independent repetitions to segregate trials into training and test subsets. At each repetition, 70% of trials (1015 trials) were randomly selected for training, and the remaining trials (435 trials) were used for the test. Then, the sub-selected bifrequency components were sorted based on their performance in decoding the fast and the slow trials in descending order. We utilized a built-in MATLAB function (*rankfeatures*, using receiver-operator-characteristic (ROC) criteria) to sort the bifrequency components. A *k*-Nearest Neighbor classifier (*k* = 1) was employed to evaluate the sorting process. This classifier assigned a query sample to the class of the single sample in the training subset that was nearest to it. We used the metric of Euclidean distance (Ed) to measure the dissimilarity between samples. The classifier was trained several times, equal to the number of bifrequency components sub-selected for each target position condition. We used the first *F* bifrequency components (features) for training the classifier, which had the top ranks in the sorting analysis. *F* was varied from 1 (the best feature) to the number of sub-selected bifrequency components (see Figures 3A,C *x*-axis). The accuracy of the classifier was assessed using the test trials. We repeated the feature-ranking method 100 times to measure the average accuracy of each *F* value. To extract the features that had better decoding performance, we set *F* based on a trade-off of maximizing two factors; (1) the ratio between the number of selected features to the total number of features (which are shown by the *x*-axes in Figures 3A,C) and (2) the decoding performance of the classifier. We extracted features for which the rank numbers were lower than *F* = 140 at each repetition. Considering this procedure, we ensured that we selected the features that provided classification accuracy above 90% in decoding the fast and the slow trials (see Figures 3B,D). Since the rank of a feature that had a moderate *F* was not consistent across different repetitions, we adopted a selection routine. This routine extracted the feature that was repeated between *F* = [1−140] across all algorithm repetitions. We applied the feature-ranking method for each target position condition (target-in\target-out, see Figure 3) and obtained 85 and 89 features (bifrequency components) for the target-in and the target-out conditions, respectively.

### Analysis of Spectral Power

We implemented a power spectrum analysis using a built-in MATLAB function (*pwelch function*). Briefly, the trial’s LFP was sub-divided into eight segments using a 500 ms time window with a 375 ms overlap. Individual segments were windowed with a Hamming window. Then, spectral density was calculated for each segment using discrete Fourier transform. The power spectrum was calculated by the average squared magnitude of spectral densities across all segments. We calculated normalized power for each trial’s LFP using the following equation:

(8) $$P\,o\,w\,e\,r=\frac{\sum_{f_{l}}^{f_{h}}P\,S\,D\,\,\left(f\right)}{\sum_{0\leq f\leq 250}P\,S\,D\,\,\left(f\right)}$$

where *f*_*l*_,*f*_*h*_ denote the lower and upper frequency bounds of the power spectral density (PSD), respectively. This equation calculates the normalized power by dividing PSD in the narrow frequency band by the total PSD. We computed PSD for frequency bands ranging between 2 and 250 Hz, with a step of 2 Hz and bandwidth of 4 Hz. This provided 124 components of normalized power for each trial’s LFP. We applied this analysis on individual trials, including all the target position conditions (target-in\ target-out).

### Analysis of Feature Extraction

**FIGURE 4:**
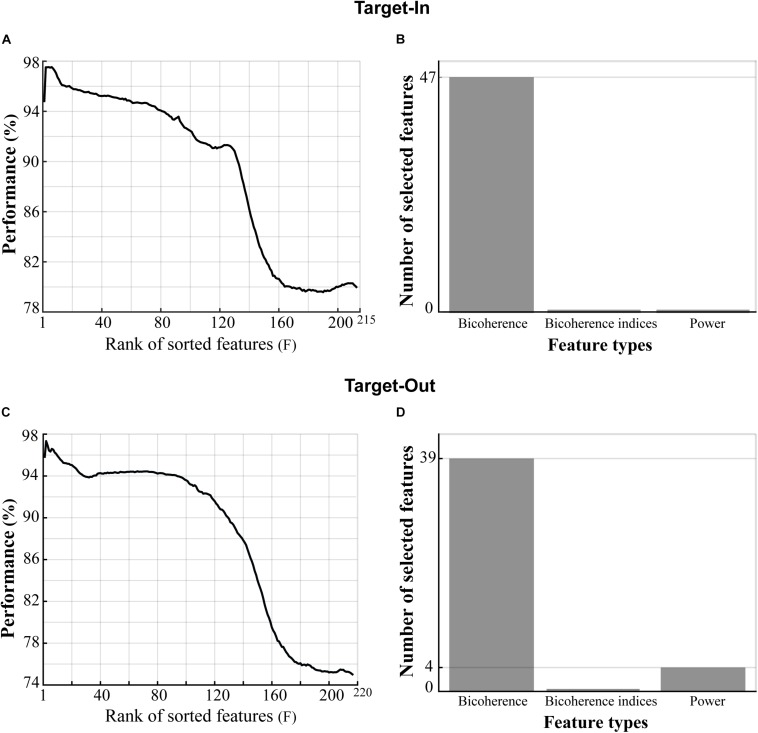
Type of features providing high classification performance in decoding the fast and the slow trials. **(A,C)** Three types of features (shown on the *x*-axis of panels **B,D**) in the Target-In and the Target-Out conditions, respectively, which were sorted based on the decoding performance in descending order. **(B,D)** Types of features in panels **(A,C)**, respectively, which provided classification performance of over 95% in decoding the fast and the slow trials. The selected bicoherence in panels **(B,D)** lies on the high-gamma frequency band (150–250 Hz, see Figure 5). The selected powers in panel **(D)** lie between 190 and 200 Hz.

We extracted three types of features from each trial’s LFP, namely: (1) the bifrequency components that were sub-selected from the bicoherence matrix, (2) the bicoherence indices, and (3) the normalized power. We used the feature-ranking method to extract the best features provided a high decoding performance for classifying the fast and the slow trials, but here, we only extracted the 60 first features (by setting *F* = 60) instead of the 140 features (*F* = 140) extracted in the original algorithm. Furthermore, we selected features that repeated across 90% of algorithm repetitions within the first 60 features (the algorithm was repeated 100 times). The classifier could reach an accuracy of over 95% in decoding the fast and the slow trials. However, despite choosing different *F* values for the analyses shown in Figures 3, 4, the number of selected features in both analyses were comparable. In more detail, about 15–30% of the total number of features were sub-selected in each analysis.

### Categorization of the Bifrequency Components

**FIGURE 5:**
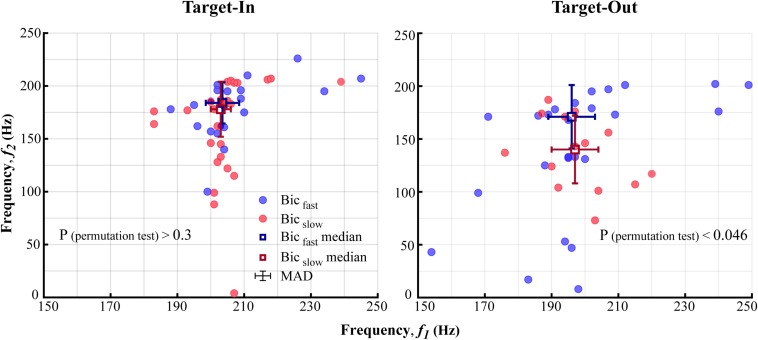
The characteristic bifrequency of QPC in fast and slow trials for each target position condition (Target-In\Target-Out). The blue (Bic_fast_) and the red (Bic_slow_) circles show the bifrequency components in which the average bicoherence was larger in fast rather than slow trials, and vice versa, respectively. Blue and red squares represent medians of Bic_fast_ and Bic_slow_groups, respectively. Error-bars demonstrate the median absolute deviation (MAD) calculated for each frequency axis. Distributions of the bifrequency component in Bic_fast_ and Bic_slow_groups are significantly different in the Target-Out condition (*p* < 0.046; using a permutation test). This is not the case for the Target-In condition (*p* > 0.3, using a permutation test).

We categorized the bifrequency components sub-selected from a trial’s feature vector into the *Bic*_*fast*_ or *Bic*_*slow*_ group based on the average bicoherence in the fast and the slow trials. To this end, a bifrequency component was labeled as *Bic*_*fast*_ or *Bic*_*slow*_ if the average bicoherence for that bifrequency component was larger in the fast than the slow trials or vice versa, respectively (Figure 5). We further calculated the median and the median absolute deviation (MAD) for each *Bic*_*fast*_ and *Bic*_*slow*_ group. The MAD is calculated using the following equation:

(9) M⁢A⁢D=m⁢e⁢d⁢i⁢a⁢n⁢⁢|X-m⁢e⁢d⁢i⁢a⁢n⁢⁢(X)|

Where *X* is a vector of the bifrequency components at each *Bic*_*fast*_ < *cps*:*it* > *or* < /*cps*:*it* > *Bic*_*slow*_group. We conducted a permutation test to analyze significant difference between bifrequency distributions of *Bic*_*fast*_ and *Bic*_*slow*_ groups. The Ed was calculated between the bifrequency components and the corresponding median at the *Bic*_*fast*_ and*Bic*_*slow*_ groups. Then, the Eds of*Bic*_*fast*_ < *cps*:*it* > *and* < /*cps*:*it* > *Bic*_*slow*_ were randomly shuffled between these groups 100,000 times. For each repetition, we calculated an absolute difference between the average Ed in the Pseudo-*Bic*_*fast *_and Pseudo-*Bic*_*slow *_group. Then, the proportion of repetitions with absolute differences larger than the original absolute difference was calculated. The proportion showed a significant difference between the bifrequency distribution of the*Bic*_*fast*_ < *cps*:*it* > *and* < /*cps*:*it* > *Bic*_*slow*_group if it was smaller than 0.05.

### Analysis of QPC Temporal Dynamic for the Fast and the Slow Trials

We employed a time window of 150 ms with a 125 ms overlap to calculate the temporal dynamic of the quadratic phase coupling (QPC) in the same analysis time window used for the original bicoherence analyses (Figure 7). We ensured that the analysis time window was at least 590 ms after the stimulus onset. Firstly, we filtered the trial’s LFP with the GWHT to calculate wavelet coefficients *a*(*f*_1_,*t*) and *a*(*f*_2_,*t*) in a given frequency pair at the *Bic*_*fast*_ or *Bic*_*slow*_ group. Second, we took advantage of the GHWT-based WBIC algorithm (Eq. 1) to compute the bicoherence in each frequency pair for each time epoch. Next, the bicoherences were averaged across frequency pairs in each *Bic*_*fast*_ and*Bic*_*slow*_ group. Eventually, we averaged the bicoherence at each time epoch for the fast and the slow trials. The following equation computes the bicoherence for each time epoch in the analysis window:

(10) $$B\,i\,c\,\left(t_{i}\right)=\frac{1}{N_{F}\times N_{T\,r}}\,\sum_{j=1}^{N_{T\,r}}\sum_{l=1}^{N_{f}}\,b_{i}^{j}\,\left(f_{1}^{l},f_{2}^{l}\right)\,i=1\,,\,2\,,\,\ldots \,,\,N_{e\,p}$$

where $b_{i}^{j}\,\left(\cdot \right)$ indicates the WBIC of  the i^*th*^ time epoch in the j^*th*^ trial, $\left(f_{1}^{l},f_{2}^{l}\right)$ shows the l^th^ frequency component in the *Bic*_*fast*_ and *Bic*_*slow*_ group, *N*_*Tr*_ denotes the total number of trials, *N*_*f*_ represents the total number of bifrequency components at each *Bic*_*fast*_ and *Bic*_*slow*_ group, and *N*_*ep*_ = 55 is the total number of time epochs in the analysis window. The *Bic*(⋅) lies between [0–1] in which zero indicates no QPC and 1 reflects perfect QPC, respectively. We used a permutation test analysis to characterize the time epochs with a significant difference between the QPC of the fast and the slow trials. The QPC of trials at each time epoch were randomly shuffled between the fast and the slow groups 1000 times. For each repetition, we calculated the difference between average QPCs in the shuffled fast and the shuffled slow trials. Then, the proportion of repetitions with larger absolute differences compared to the original absolute difference was calculated. The time epochs that had a proportion smaller than 0.05 were considered the time epoch with a significant difference between QPCs in the fast and the slow trials. We next used false discovery rate (FDR) for multiple comparisons.

### Analysis of Correlation Between Single-Unit Spiking Activity and Bicoherence

**FIGURE 6:**
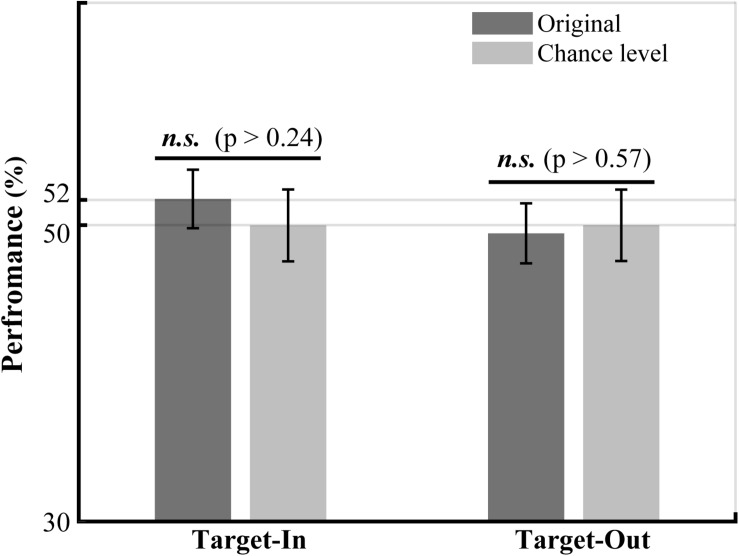
The performance of LFP power in the high-gamma frequency band (190–210 Hz) in decoding the fast and the slow trials. The LFP power in the high-gamma frequency band was calculated for the fast and the slow trials using the Welch method (see Materials and methods). The bounds of high-gamma frequencies (i.e., 190 to 210 Hz) were defined based on the frequency ranges in Figure 5 with a high concentration of bifrequency components. We normalized the high-gamma power to the average high-gamma power at each site. The performance of the high-gamma power for decoding the fast and the slow trials was calculated by employing a *k*-Nearest Neighbor classifier. We used a repeated holdout method (100 times) to subdivide the trials into the training and the test subsets. In each repetition, we used 70% of the trials for training and the remaining trials for the test. The chance level was calculated for each target position condition by repeatedly (100 times) shuffling the trials between the fast and the slow groups. The result clearly indicates that the decoding performance of high-gamma power is not significantly different between the original and the shuffled high-gamma power in each target position condition (permutation test, *p* > 0.05). The *p*-values have been shown on top of the bars.

We pooled the fast and the slow trials, regardless of their behavioral outcomes. First, we calculated the bicoherence at each bifrequency component in the *Bic*_*fast*_ and*Bic*_*slow*_ group to measure correlation between single-unit spiking activity and bicoherence. Second, the bicoherence at each bifrequency component was *z*-score-normalized across trials. Third, the trial’s bicoherence was averaged across all bifrequency components at each *Bic*_*fast*_ and *Bic*_*slow*_ group. Fourth, we calculated the trial’s spike-rate using single-unit spiking activity. The analysis of spike-rate was conducted for the same time window used in the bicoherence analyses. Fifth, the Spearman’s correlation was employed to calculate the correlation between the trial’s spike-rate and the trial’s bicoherence at each *Bic*_*fast*_and *Bic*_*slow*_ group (see Figures 6, 8).

**FIGURE 7:**
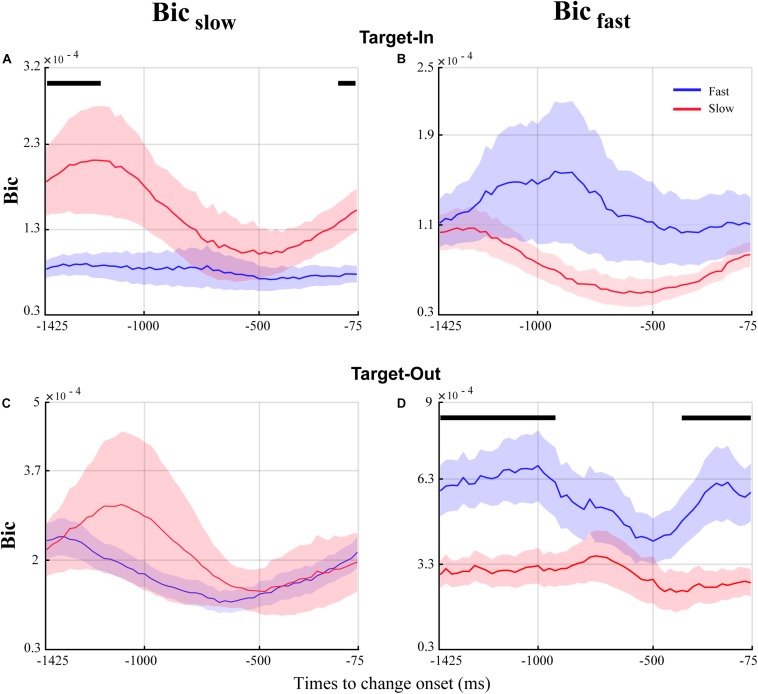
Temporal dynamics of QPC in the fast and the slow trials for the time window before the target change. The blue and red curves show the temporal dynamics of QPC for the fast and the slow trials, respectively. The curves show the average bicoherence for the bifrequency component of the _Bicfast_(right column) and Bic_slow_(left column) groups shown in Figure 5. The black lines on top of the traces mark the times that the QPC of the fast and the slow trials are significantly different (*p* < 0.05, permutation test, FDR corrected for multiple comparisons).

**FIGURE 8:**
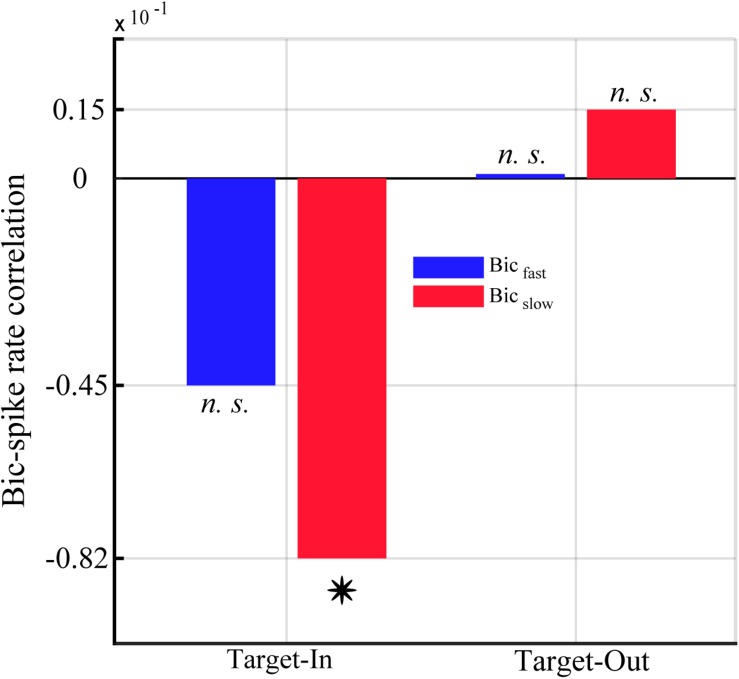
Correlation between the single-unit spike rate and the QPC of characteristic bifrequencies in Bic_fast_and Bic_slow_ groups for the Target-In and the Target-Out condition. *Y*-axis represents the strength of correlation between the QPC and the spike rate using Spearman’s correlation method. The star shows a significant correlation in Bic_slow_ groups at the Target-In condition (*p* < 0.002, Spearman correlation).

## Results

To study the functional interaction of neural circuits underlying behavior, we trained a monkey to perform a change detection task. In brief, the monkey had to covertly attend to one (target) of two coherently random dot patterns (RDP). The monkey was rewarded with a drop of juice if it correctly detected a short direction change in the target RDP (Figure 1, see also section “Materials and Methods”). The monkey correctly reported the target change in 86% of trials without breaking its eye fixation. We recorded the local field potentials (LFP) and the single-unit spiking activity from the MT area while the monkey performed the task. To study the neural process underlying behavior, we analyzed the hit trials in which the monkey correctly detected the target change. The hit trials were subdivided into fast and slow trials based on the animal’s reaction time (see section “Materials and Methods”). Next, we calculated the bicoherence for LFPs to investigate how the non-linear neuronal synchronization likely leads to fast or slow behavior. We employed general harmonic wavelet transform (GHWT)-based wavelet bicoherence (WBIC) (Li et al., 2009, 2011) to measure the strength of QPC in LFP signals. We applied GHWT-based WBIC for a time window of 1500 ms before the target change occurred. Our analyses indicated that the QPC, especially in high-gamma frequencies (150–250 Hz), can reliably decode the animal’s reaction time. Moreover, we observed that the characteristic frequency pair of QPC are selective to the target position condition (target-in\target-out) and the speed of visuomotor behavior (fast\ slow).

### QPC Influences Behavior Systematically

Analysis of QPC allows us to measure the phase synchrony between three signals with different frequencies. We applied the GHWT-based WBIC method on LFPs to calculate the bicoherence for each trial. We calculated the bicoherence for all frequency pairs [e.g.,(*f*_1_,*f*_2_)] between 1 and 250 Hz, with a step of 1 Hz and bandwidth of 2 Hz. This provided a two-dimensional matrix (bicoherence matrix) with 250 × 250 components for each trial. Each element in the bicoherence matrix represents the strength of QPC in a pair of frequency components (bifrequency) in the LFP spectrum. To analyze the bicoherence matrix, we calculated four indices (Li et al., 2009, 2011, 2013; Wang et al., 2017), namely (I) total bicoherence (Total Bic), (II) average diagonal elements (Diagonal Bic), (III) maximum eigenvalue, and (IV) Shannon entropy of the eigenvalue distribution (see section “Materials and Methods”). Figure 2 shows the bicoherence indices in the fast and the slow trials (blue and red, respectively) for the target-in and target-out condition. The result clearly demonstrates that the strength of QPC in fast trials is significantly larger than in slow trials for the three bicoherence indices and both target position conditions (*p* < 0.03, using two-sided Wilcoxon rank-sum test, excluding the significance level in the Shannon entropy of eigenvalues). In addition, these differences in bicoherence indices are not due to the difference between the length of stimulus presentation in the fast and the slow trials (see Supplementary Figure S1). Moreover, it is visually evident that the strength of bicoherence indices is clearly enhanced in the target-in condition compared with the target-out condition, irrespective of the animal’s reaction time. This observation suggests that a cognitive process like selective attention probably modulates the QPCs in the target-in condition. However, the QPC enhancement in fast trials among both target position conditions reflects that the QPC is potentially not a cortical function that is preferably processing only that stimulus placed inside the neuron’s RF.

### QPC in High-Gamma Frequencies Plays a Crucial Role in Guiding Behavior

We next examined how neuronal oscillatory activities at different frequencies can individually or interactively contribute to the processing of visuomotor information in MT cortex. For this purpose, we analyzed the bicoherence matrix to find the bifrequency component that provided maximum discrimination between the fast and the slow trials. Firstly, we extracted the bifrequency components that showed a significant difference between the fast and the slow trials (*p* < 0.01using two-sided Wilcoxon rank-sum test). Then, a feature ranking method was employed for sub-selecting the bifrequency components provided the classification performance above 90% in decoding the fast and the slow trials (see section “Materials and Methods” for more details). Figures 3A,C show the bifrequency components (features) sorted based on their decoding performances in descending order in the target-in and the target-out condition, respectively. Figures 3B,D demonstrate the distributions of the bifrequency components for the features in Figures 3A,C, respectively, showing a decoding performance above 90%. There are 85 and 89 bifrequency components in Figures 3B,D, respectively. It is visually evident that the selected bifrequency components are distributed in a broadband high-gamma frequency range for each target position condition (100–250 Hz). In addition, the result clearly indicates that the selected bifrequency components in the target-out condition are more widely distributed than the target-in condition in the high-gamma frequency range.

We next examined the contributive role of the spectral power to the bicoherence in decoding the animal’s RT. The idea is that the power spectrum does not retain the phase information of the signal but captures the statistical property of the signal’s Gaussianity. In contrast, the bicoherence can extract information relevant to the signal’s non-Gaussianity and signal phase spectrum (Nikias and Mendel, 1993). We computed LFP power in narrow frequency bands between 2 to 250 Hz, with a step of 2 Hz and bandwidth of 4 Hz. Then, the LFP powers were normalized to the total power in 2–250 Hz *(see materials and methods)*. Next, we defined a feature vector for each trial including three types of features: (I) the selected bifrequency components shown in Figures 3B,D, (II) the normalized spectral powers, and (III) the bicoherence indices (see section “Materials and Methods”). We employed the same selection routine used for Figures 3B,D to select the best feature from the feature vector. In brief, we sorted the features based on decoding performances in descending order. We then extracted features yielding classification performance of over 95% in decoding the fast and the slow trials (see section “Materials and Methods” for more details). Figures 4A,C show the sorted features in the target-in and the target-out conditions, respectively. Figures 4B,D demonstrate the types of selected features extracted from Figures 4A,C, respectively. The result is clearly evident that bicoherence is the most frequent type of feature that was selected for each target position condition. This observation suggests that QPC in the high-gamma frequency band functionally plays a key role in guiding the fast and the slow behavioral responses.

### Switching Toward the Neuron’s RF Modulates the Characteristic Bifrequency of the Fast and the Slow Trials

To further investigate how the selected bifrequency components in Figures 4B,D are distributed across the bifrequency map, we subdivided the bifrequency component into *Bic*_*fast*_ < *cps*:*it* > *and* < /*cps*:*it* > *Bic*_*fast*_groups based on average bicoherence in the fast and the slow trials. In more detail, a bifrequency component was labeled as *Bic*_*fast*_ or *Bic*_*slow*_ if the its average bicoherence was larger in the fast compared to the slow trials or vice versa, respectively. We further calculated the median and the median absolute distance (MAD) for the bifrequency component of the *Bic*_*fast*_ < *cps*:*it* > *and* < /*cps*:*it* > *Bic*_*fast*_group per target position condition (see section “Materials and Methods”). Figures 5 and Supplementary Figure S2 show the distribution of bifrequency components in *Bic*_*fast*_ < *cps*:*it* > *and* < /*cps*:*it* > *Bic*_*slow*_ groups for each position condition. The result indicates that the QPC in a narrower band of high-gamma frequencies (i.e., 150–250 Hz, instead of 100–250 Hz in Figures 3B,D) is more implicated in guiding visuomotor behavior. In addition, the high-gamma QPC is disassociated from potential differences between high-gamma powers in the fast and the slow trials (see Figure 6). The result demonstrates that distributions of the bifrequency in*Bic*_*fast*_ < *cps*:*it* > *and* < /*cps*:*it* > *Bic*_*slow*_ groups are not significantly different for the target-in condition (*p* > 0.3, using a permutation test, see section “Materials and Methods”) and show a significant difference for the target-out condition (*p* < 0.046, using a permutation test, see section “Materials and Methods”). Given that *Bic*_*fast*_ < *cps*:*it* > *and* < /*cps*:*it* > *Bic*_*slow*_ potentially represent the characteristic bifrequency of QPC in the fast and the slow trials, respectively, the result visually indicates that the distributions of characteristic bifrequencies in the fast and the slow trials are clearly different across target position conditions. In more detail, we observe that the medians of characteristic bifrequency in *Bic*_*fast*_ and *Bic*_*slow*_ groups increases for (7 Hz, 7 Hz) and (6 Hz, 38 Hz) for each (*f*_*1*_, *f*_2_) dimension, respectively, when the monkey performed the target-in condition. In addition, the result clearly illustrates that the median of characteristic bifrequency in slow trials is strongly modulated by switching toward the neuron’s RF (i.e., target-in condition). But this is not the case for the fast trials. Despite this observation, the increase of medians in the target-in condition suggests that the frequency of non-linear coupling increases when the monkey attends to the stimulus inside the neuron’s RF. Despite the different distributions of the characteristic bifrequencies in fast and slow trials across target position conditions, the decoding performance of QPC is similar across the target-in and the target-out conditions (see Figures 3B,D; see section “Materials and Methods”).

### QPC of Characteristic Bifrequencies in the Fast and the Slow Trials Follow Different Temporal Dynamics Among Target Position Conditions

Our analyses highlighted that the QPC in the high-gamma frequency band plays a crucial role in processing visuomotor information. In addition, we observed that the fast and the slow trials are discriminated by the distinct characteristic bifrequency of QPC in the MT area. To study the time dynamics of QPC in the fast and the slow trials, we used a time window of 150 ms with a 125 ms overlap. We calculated the bicoherence at each characteristic bifrequency of*Bic*_*fast*_ and*Bic*_*slow*_ for a given time window. Then, the bicoherence was averaged across all characteristic bifrequencies and trials for each time window (see section “Materials and Methods” for more details). Figure 7 shows the temporal dynamics of QPC in the fast and the slow trials using characteristic bifrequncies of Bic_*fast*_ and*Bic*_*slow*_ groups in each target position condition. We observed that the QPC in the fast and the slow trials follows a distinct temporal pattern at each target position condition using characteristic bifrequencies of Bic_*fast*_ and*Bic*_*slow*_ groups. In addition, it is visually evident that the magnitude of the significant QPC difference between the fast and the slow trials in Figure 7C is somewhat enhanced in Figure 7A (comparing the times showing a significant difference between red and blue curves in Figure 7A with the corresponding times in Figure 7C). In other words, the significant QPC difference between fast and slow trials in characteristic bifrequencies of Bic_*slow*_ is modulated when the monkey attends to the stimulus inside the neuron’s RF. In contrast, we observe that the magnitude of the significant QPC difference between the fast and the slow trials in Figure 7D strongly decreases in Figure 7B (comparing the times showing a significant difference between the red and blue curves in Figure 7D with the corresponding times in Figure 7B). In other words, the significant QPC difference between fast and slow trials in the characteristic bifrequencies of Bic_*fast*_ strongly decreases when the animal attends to the stimulus inside the neuron’s RF. We hypothesize that these contrary observations in the characteristic bifrequency of the fast and the slow behavior (i.e., Bic_*fast*_ and*Bic*_*slow*_, respectively) along target position conditions are potentially due to the influence of a cognitive function like attention. Our hypothesis is in line with previous studies that have shown that attention could decouple sensory neurons and thereby enhance the neural representation of relevant stimuli to effectively guide a fast behavioral reaction (Esghaei et al., 2015, 2018; Spyropoulos et al., 2018).

### QPC Is Anti-correlated With the Neuronal Spike Rate Exclusively for the Target-in Condition

We calculated the correlation between the QPC and the single-unit spiking activities to study how neuronal non-linear coupling potentially influences the neuronal output (see section “Materials and Methods”). We computed the single-unit spike rate as well as the average normalized QPC for each trial using characteristic bifrequencies of *Bic*_*fast*_ < *cps*:*it* > *and* < /*cps*:*it* > *Bic*_*slow*_ groups (shown in Figure 5). Figure 8 shows the Spearman correlation between spike rates and QPCs in each target position condition. The result indicates that the QPC and the spike rate are strongly anti-correlated in the target-in condition. Notably, we observe that the spike rates and the QPC of the *Bic*_*slow*_ group are significantly anti-correlated in the target-in condition (*p* < 0.002, using Spearman correlation). In contrast, there is a negligible non-significant positive correlation between QPCs and the spike-rates for the target-out condition.

## Discussion

Many studies have highlighted that oscillatory activity plays a mediating role in the neuronal coupling underlying cognitive functions (Lisman and Jensen, 2013; Khodagholy et al., 2017; Rohenkohl et al., 2018). However, the relationship between this neuronal coupling and behavior has not been studied in the visual cortex.

In this study, we recorded the LFP and the single-unit spiking activity from the visual area MT of a behaving monkey. The animal had to covertly attend to one of two RDPs placed inside or outside the RF of recorded neurons and detect a short direction change in the target stimulus. We examined how linear and non-linear neural synchronization could influence the animal’s RT. For this purpose, the spectral representation of the second-order statistics (i.e., the power spectrum) and the third-order statistics (i.e., bicoherence) were calculated for LFPs on a trial-by-trial basis.

We measured the strength of non-linear coupling between all frequency pairs in the LFP spectrum (1–250 Hz) using four bicoherence indices. The bicoherence indices were: (i) total Bic, which reflects the strength of QPC between different low-frequency oscillations and one of the high-frequency oscillations, which is useful for investigating the strength of rhythmic synchronization between neuronal populations oscillating at different frequencies (Li and Li, 2016), (ii) maximum eigenvalue, (iii) Shannon entropy of eigenvalues, which measures information on the synchronization between oscillatory activities in the neuronal population (Li and Li, 2016), and (iv) diagonal Bic, which reveals the presence of self-frequency and self-phase coupling in neural circuits (Muthuswamy et al., 1999). We selected the trials which had no significant difference between average spectral powers to prevent dependence of our analyses to the different levels of 1/*f* noise (Bédard et al., 2006; Lombardi et al., 2017). With this approach, we ensured that the signal-to-noise ratio (SNR) was not significantly different between the chosen fast and slow trials (see section “Materials and Methods”). In addition, we ensured that the change in the bicoherence indices was potentially due to the change in underlying neuronal non-linear coupling (Pesaran et al., 2018). Our analysis revealed that the strength of the non-linear coupling between the oscillatory activities of MT neurons is strongly increased in the fast rather than the slow trials (see Figure 2). Furthermore, we observed that switching toward the neuron’s RF increases the strength of non-linear coupling between neural oscillations. We speculate that this finding is possibly due to the influence of a cognitive function like attention that enhances the non-linear synchronization between local neurons. Our hypothesis is in line with previous studies that have shown that spatial attention selectively increases the strength of synchronization between neurons processing the target stimulus (Womelsdorf et al., 2006; Zareian et al., 2018; Khamechian et al., 2019).

To further study the non-linear neuronal synchronization underlying behavior, we implemented a machine learning approach to extract the bifrequency component that accurately discriminates the fast and the slow trials (see section “Materials and Methods”). The result showed that oscillatory activities in the high-gamma frequency band (100–250 Hz) are quadratically phase-coupled in the fast and the slow trials (Figures 3B,D). This observation is in line with a recent study showing that the strength of neural synchronization in the high-gamma frequency band (180 to 220 Hz) predicts the animal’s RT (Khamechian et al., 2019). In addition, this study also showed that the difference between high-gamma synchronizations in the fast and the slow trials cannot be attributed to the difference between the magnitude of the spike leakage onto LFPs (Khamechian et al., 2019). Many studies have suggested that interneurons contribute to the generation of high-gamma oscillations in the LFPs (Brunel and Wang, 2003; Buzsáki and Draguhn, 2004; Henrie and Shapley, 2005; Gieselmann and Thiele, 2008; Stark et al., 2014; Suffczynski et al., 2014).

We next examined the contributive role of cortical Gaussian and non-Gaussian processes (activities) in guiding visuomotor behavior. We computed the power spectrum (as a measure of Gaussianity) and the bicoherence (as a measure of non-Gaussianity) for the fast and slow the trials. We then adopted a machine learning method (see section “Materials and Methods”) to examine the potential role of these processes in predicting the animal’s behavior. The results illustrated that the neural non-Gaussian process (in addition to the Gaussian process) plays a key role in coding behavioral RTs in the macaque area MT (see Figures 4B,D). The result is consistent with a recent study indicating that bicoherence is a biomarker candidate for identifying neurodevelopmental-behavioral disorders like attention deficit hyperactivity disorder (ADHD) (Chen et al., 2019).

We further examined the QPC to understand which specific bifrequency of the bicoherence at the broadband high-gamma frequency range (100–250 Hz, see Figure 3) might orchestrate the fast and the slow behaviors. The result indicated that the distribution of the characteristic bifrequency is significantly different between the fast and the slow trials, particularly for the target-out condition (see Figure 5). In addition, we observed that switching toward the neuron’s RF enhances the characteristic bifrequency of the QPC explicitly in the slow trials. We speculate that a top-down cognitive function like attention probably modulates the characteristic bifrequency of the QPC in slow trials in the target-in condition. In addition, our observations suggest that this modulatory effect mostly occurs between the MT neurons that selectively process the target stimulus. Moreover, we observed that the characteristic bifrequency of the fast trials has similar distribution medians in the target-in and the target-out conditions. The result suggests that entire neurons in the MT area can be synchronized in the high-gamma band to efficiently process the behavioral information and facilitate a fast behavioral action. Given that spatial attention can effectively shorten RT (Posner, 1980; Womelsdorf et al., 2006) and modulate neuronal synchronization (Womelsdorf et al., 2006; Hoogenboom et al., 2010; Khamechian et al., 2019), we hypothesize that such synchronization in the fast trial can also be attributed to top-down attention. In addition, our hypothesis is in line with previous studies that suggested that attention could improve neuronal communication and thereby route the most relevant information into associative areas in the brain (Gregoriou et al., 2009; Morishima et al., 2009; Briggs et al., 2013).

We next examined the temporal dynamics of QPC in the fast and the slow trials based on the characteristic bifrequency obtained for each target position condition. The result demonstrated that switching to the neuron’s RF enhances the QPC difference between the fast and the slow trials using the characteristic bifrequency of the slow behavior (i.e.,*Bic*_*slow*_). In contrast, we observed that the QPC difference between the fast and the slow trials strongly decreases in the characteristic bifrequency of the fast behavior (i.e., Bic_*fast*_) when the monkey covertly attends to the stimulus inside the neuron’s RF. We hypothesize that this contrary observation for the QPC difference in the characteristic bifrequency of the fast and the slow behavior is due to a cognitive function like attention. Our speculation is based on previous studies suggesting that attention can suppress the strength of coupling between oscillatory activities in the visual cortex (Esghaei et al., 2015; Spyropoulos et al., 2018).

Some physiological models have shown that decisions are formed based on accumulating sensory evidence over time to a bound (Gold and Shadlen, 2001; Palmer et al., 2005; Ratcliff and McKoon, 2008). In addition, they have indicated that these computations could shape the RT distribution and the speed of behavior. The accumulation of evidence has been observed in several electrophysiological studies at different cortical areas of monkeys (Roitman and Shadlen, 2002; Purcell et al., 2012; de Lafuente et al., 2015), rodents (Hanks et al., 2015), and humans (Kelly and O’Connell, 2013; Twomey et al., 2016). For example, some of these studies reported that oscillatory activities underlying the accumulation process follow different accumulation-to-bound dynamics that predict the behavioral RTs (Kelly and O’Connell, 2013; Twomey et al., 2016). However, it is unclear how these oscillatory activities transmit sensory information from upstream to downstream cortical areas to shape the accumulation process. Previous investigations have shown that neural oscillatory activities can interact via CFC to facilitate communication of information between brain regions (Darvas et al., 2009; Canolty and Knight, 2010; Holz et al., 2010; Fiebelkorn et al., 2018). Based on these studies, we speculate that the QPC (as a non-linear form of CFC measured by the bicoherence) could play a functional role in the transmission of the relevant information between associative neurons in the intra- or inter-areal of the cortex.

In summary, we employed bicoherence and spectral power to examine non-linear and linear neuronal coupling underlying visuomotor behavior. Our results show that: (I) the non-linear phase coupling between oscillatory activities of sensory neurons is a good candidate for predicting the speed of the animal’s behavior, (II) the non-linear neuronal coupling is expressed in a broad band of high-gamma frequencies (100–250 Hz) in area MT of the macaque visual cortex, (III) the non-Gaussian cortical process (measured by the bicoherence) and the Gaussian process (measured by the spectral power) are both involved in the processing of visuomotor information, and (IV) the non-linear characteristic of neuronal synchronization among MT neurons is probably controlled by a cognitive function like selective attention.

## Data Availability Statement

The datasets analyzed in this manuscript are not publicly available. Requests to access the datasets should be directed to MD, mailto:daliri@iust.ac.ir (upon reasonable request).

## Ethics Statement

The animal study was reviewed and approved by the responsible regional government office [Niedersaechsisches Landesamt fuer Verbraucherschutz und Lebensmittelsicherheit (LAVES)] under the permit numbers 33.42502/08-07.02 and 33.14.425 02-04-064/07.

## Author Contributions

MK and MD designed the study, interpreted the data and wrote the manuscript. MD recorded the data. MK performed the data analyses.

## Conflict of Interest

The authors declare that the research was conducted in the absence of any commercial or financial relationships that could be construed as a potential conflict of interest.
