# Supplementary material for: Decoding Adaptive Visuomotor Behavior Mediated by Non-linear Phase Coupling in Macaque Area MT
Source: Front Neurosci. 2020 Apr 3;14:230. doi: 10.3389/fnins.2020.00230 (PMC7147352; doi:10.3389/fnins.2020.00230)
Supplement: Supplementary file 1 [file Presentation_1.pdf]

Supplementary Material

Decoding adaptive visuomotor behavior mediated by nonlinear phase coupling in macaque area MT

Mohammad Bagher Khamechian, Mohammad Reza Daliri*

*corresponding author:

Mohammad Reza Daliri (Email: daliri@iust.ac.ir)

Supplementary Figures

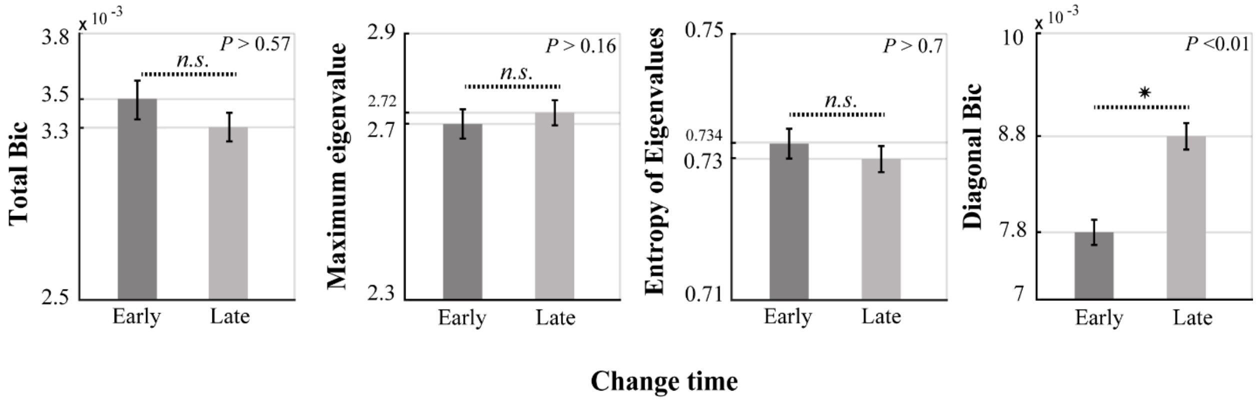

Supplementary Figure S1. Bicoherence indices for the trials that had early and late target-change events. The fast and the slow trials in the target-in and the target-out condition were pooled and sorted based on the length of stimulus presentation. To select two subsets of trials with maximum differences in length of stimulus presentation, we partitioned the sorted trials into six subsets and selected the first and the last partitions as early and late change trials. Obviously, these subsets of trials were significantly different in the length of stimulus periods ($p < 10^{-154}$, using two-sided Wilcoxon rank-sum test). Each selected partition contained at least 460 trials. Then, we calculated the bicoherence indices in the same time windows used for the original bicoherence analyses (see “Analysis window” in Fig. 1). The result clearly shows that the bicoherence indices are not reliable predictors for the length of stimulus presentations.

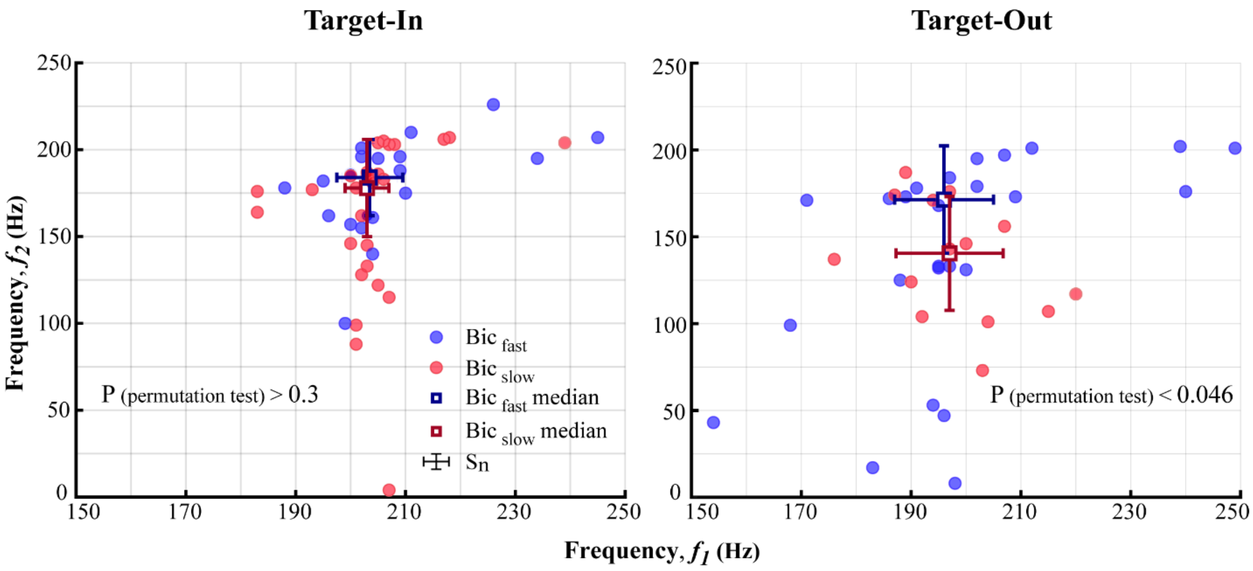

Supplementary Figure S2. The characteristic bifrequency of QPC in the fast and the slow trials for each target position condition (Target-In\Target-Out). This figure is a replication of Fig. 5, but the error-bars were calculated using the scale estimator "S_n" method. The scale estimator is an alternative approach to MAD (used in Fig. 5) that is employed for skewed distributions (Rousseeuw and Croux, 1993). "S_n" is computed by $S_n = \text{median}_i\{\text{median}_j\left(\left|x_i - x_j\right|\right)\}$, where i and j are two arbitrary samples from a total of n samples in a given distribution (i.e., Bic_{fast} or Bic_{slow}).

Supplementary References

Rousseeuw, P. J., and Croux, C. (1993). Alternatives to the Median Absolute Deviation. *J. Am. Stat. Assoc.* 88, 1273–1283. doi:10.1080/01621459.1993.10476408.
